# Virulent strains of *Pseudomonas aeruginosa* are more disruptive during invasion of a microbial model community

**DOI:** 10.1093/femsle/fnag090

**Published:** 2026-07-31

**Authors:** Emil Burman, Mikkel Anbo, Lars Jelsbak, Johan Bengtsson-Palme

**Affiliations:** Division of Systems and Synthetic Biology, Department of Life Sciences, SciLifeLab, Chalmers University of Technology, SE-412 96 Gothenburg, Sweden; Department of Infectious Diseases, Institute of Biomedicine, Sahlgrenska Academy, University of Gothenburg, Guldhedsgatan 10, SE-413 46 Gothenburg, Sweden; Centre for Antibiotic Resistance Research (CARe) in Gothenburg, Sweden; Department of Biotechnology and Biomedicine, Technical University of Denmark, Søltofts Plads, Building 221, DK-2800, Kongens Lyngby, Denmark; Department of Biotechnology and Biomedicine, Technical University of Denmark, Søltofts Plads, Building 221, DK-2800, Kongens Lyngby, Denmark; Division of Systems and Synthetic Biology, Department of Life Sciences, SciLifeLab, Chalmers University of Technology, SE-412 96 Gothenburg, Sweden; Department of Infectious Diseases, Institute of Biomedicine, Sahlgrenska Academy, University of Gothenburg, Guldhedsgatan 10, SE-413 46 Gothenburg, Sweden; Centre for Antibiotic Resistance Research (CARe) in Gothenburg, Sweden

**Keywords:** microbiomes, bacterial competition, community interactions, model microbial communities

## Abstract

*Pseudomonas aeruginosa*, a major opportunistic pathogen involved in cystic fibrosis and many nosocomial infections, exhibits strong competitive abilities in microbial communities. However, the mechanisms underlying its competitive success remain poorly understood. To investigate this, we introduced two *P. aeruginosa* strains—the moderately virulent PAO1 and the highly virulent PA14—into the THOR (The Hitchhikers Of the Rhizosphere) microbial model community, consisting of *Pseudomonas koreensis, Flavobacterium johnsoniae*, and *Bacillus cereus*. Our results show that *P. aeruginosa* invasion significantly disrupts THOR biofilms, with greater reductions in biofilm biomass and shifts in microbial composition observed with PA14 compared to PAO1. This suggests that virulence traits expressed by *P. aeruginosa* play critical roles also in its ability to outcompete resident microbes. Additionally, we examined the competitive impact of an O-antigen-deficient *P. aeruginosa* strain, showing further reduced competitive ability with other community members. While our model system has limitations in replicating host-associated environments, it provides valuable insights into *P. aeruginosa*’s ecological strategies, with implications for understanding its competitive behaviour in community settings.

## Teaser

Discover how Pseudomonas aeruginosa's virulence traits empower it to dominate microbial communities and disrupt biofilms – insights that could reshape strategies against infections.

## Introduction

*Pseudomonas aeruginosa*, a prevalent nosocomial Gram-negative pathogen, is a leading cause of chronic, severe pneumonia, particularly in patients with cystic fibrosis (Bodey et al. 1983). Owing to a robust capacity for biofilm formation, *P. aeruginosa* also causes a wide range of persistent infections, including catheter-associated urinary tract infections, bloodstream infections, and endocarditis. In these patients with cystic fibrosis, *P. aeruginosa* typically dominates the lung microbiota, contributing to disease severity (Acosta et al. 2020). Notably, greater microbiome disruption in these patients correlates with more severe clinical outcomes (Malhotra et al. 2019), suggesting that the pathogen’s competitive interactions within microbial communities may mirror mechanisms that drive its virulence. However, the ways in which *P. aeruginosa* successfully competes with a microbial community remains poorly understood. To address this gap of knowledge, community-invader experiments are necessary. However, due to the complexity of natural microbial communities, it is hard to make mechanistic inferences regarding competition strategies. Hence, ex vivo microbial model systems, such as microbial model communities, have been proposed as alternatives to natural systems (Bengtsson-Palme 2020). These communities consist of defined microbial strains that engage in quantifiable antagonistic and synergistic interactions which determine the community’s stable state. One such model community is THOR (The Hitchhikers Of the Rhizosphere), which comprises three members: *Pseudomonas koreensis, Bacillus cereus*, and *Flavobacterium johnsoniae* (Lozano et al. 2019). THOR is characterized by several measurable interactions, including intrinsic biofilm formation (Burman and Bengtsson-Palme 2021), elevated counts of *P. koreensis* within biofilms, and *F. johnsoniae*-mediated peptidoglycan production that supports *B. cereus* growth in nutrient-poor root exudate. Furthermore, *P. koreensis* secretes koreenceine, an alkaloid antibiotic that suppresses *F. johnsoniae* growth (Hurley et al. 2022), an effect neutralized by *B. cereus* through the action of the *spo0H* genetic cluster (Lozano et al. 2019).

Although THOR originates from the rhizosphere rather than the human microbiome, it captures several ecological principles that are directly relevant for understanding pathogen invasion in polymicrobial communities. The THOR members engage in biofilm formation, metabolic cross-feeding, niche partitioning, and contact-dependent antagonism, features that are also characteristic of human-associated communities (Culp and Goodman 2023). Importantly, THOR provides a stable, tractable, and experimentally reproducible model in which interspecies interactions are well defined and quantitatively measurable. This simplifies the mechanistic dissection of how an invading pathogen perturbs the resident community and enables investigation of general principles of microbial competition and displacement that extend beyond plant-associated systems. Thus, while not intended to recapitulate the full complexity of a human commensal microbiota, THOR offers a mechanistically rich and controlled context to probe how pathogens establish themselves within pre-existing microbial ecosystems.

THOR has previously been used to quantify microbial interactions and how they shift in response to external perturbations (Burman and Bengtsson-Palme 2021). However, its potential as a probe to investigate interactions between invaders and microbial communities remains unexplored. To determine if *P. aeruginosa*’s ability to occupy and disrupt microbial communities is dependent on its virulence status, we invaded established THOR biofilms with the moderately virulent PAO1 and the highly virulent PA14 strains (He et al. 2004, Lee et al. 2006, Mikkelsen et al. 2011, Grace et al. 2022).

Given the capacity of *P. aeruginosa* to invade biofilms across diverse ecological niches and naturally occurring biofilms, we hypothesized that its invasion will decrease biofilm production of the THOR microbial model community. Furthermore, since dominance of natural communities in human patients by *P. aeruginosa* is associated with more virulent strains, we hypothesized that those strains would be more dominant in a model microbial community setting as well.

We found that the quantifiable interaction networks within the THOR microbial model community were disrupted by the invasion of *P. aeruginosa*, with the extent of disruption being connected to the virulent background of the invading *P. aeruginosa*. These findings suggest that *P. aeruginosa* virulence plays a critical role in the competition between this opportunistic pathogen and surrounding microbial communities.

## Methods

### Bacterial strains and growth conditions

The bacterial strains *Pseudomonas koreensis* (CI12), *Flavobacterium johnsoniae* (UW101), and *Bacillus cereus* (UW85), collectively referred to as THOR, were obtained from the Handelsman Lab (University of Wisconsin-Madison, USA) (Handelsman et al. 1990, Lozano et al. 2019). *Pseudomonas aeruginosa* strain PA14 (DSM 19 882) was purchased from DSMZ (Deutsche Sammlung von Mikroorganismen und Zellkulturen-Leibniz Institut), PAO1 and the derivative PAO1ΔOSA strain of *P. aeruginosa* was provided and constructed by Anbo and colleagues (Anbo and Jelsbak 2023, Anbo et al. 2024). mPAO1 was acquired from Manoil lab, at university of Washington, Washington (Held et al. 2012). CCUG73745 and CCUG71613 were acquired from the Cell Culture Collection of the University of Gothenburg (CCUG) Strains were kept in −80°C in LB-glycerol and were revived by streaking onto Luria Bertani (LB) agar plates (Sigma–Aldrich, L2897). *P. koreensis* and *B. cereus* were incubated at 28°C for 14–18 h, whereas *F. johnsoniae* was incubated at 28°C for 36–48 h. After incubation, plates were stored at 4°C for a maximum of 7 days prior to the preparation of liquid cultures. Liquid cultures were prepared by inoculating single colonies into 15 ml conical tubes (VWR, 525–0604) containing 3 ml of 50% strength Tryptic Soy Broth (TSB) and incubating at 28°C for 14–18 h under constant shaking at 150 rpm. All *Pseudomonas aeruginosa* strains were maintained on LB agar plates, incubated at 37°C for 14–18 h, and stored at 4°C for up to 7 days. Plates exceeding this storage period were discarded, and fresh cultures were prepared as needed.

Strain isolates and their sources, together with the associated references are listed in Supplementary Table S1.

### THOR community establishment

Overnight cultures of *P. koreensis, F. johnsoniae*, and *B. cereus* were diluted in 10% Tryptic Soy Broth (TSB) to achieve a final optical density at 600 nm (OD₆₀₀) of 0.004 (∼10^4^ CFU/ml) for *P. koreensis* and 0.001 (∼10⁶ CFU/ml) for *F. johnsoniae* and *B. cereus*. The diluted cultures were transferred to 96-well plates (Sarstedt: 83.3924) and incubated at 18°C for 15 h. Overnight cultures of *P. aeruginosa* wild-type strains were diluted to a final OD₆₀₀ of 0.08 (∼10⁵ CFU/ml). A 5 µl aliquot of these cultures was added to each well containing the THOR co-cultures. The plates were incubated for an additional 24 h at 18°C. Biofilms were harvested by washing each well three times with 200 µl of 1 × phosphate-buffered saline (PBS). The liquid-air interface of each well was scraped using a sterile micropipette tip, and the biofilm solution was transferred to a new 96-well plate. Serial 10-fold dilutions (seven levels) were prepared from each sample. The dilution series was then plated onto species selective LB-agar. Specifically, we used the following combinations of species and antibiotics: *P. aeruginosa*—Irgasan (25 µg/ml), *B. cereus*—Polymyxin B (5 µg/ml), *F. johnsoniae*—Gentamycin (10 µg/ml), and *P. koreensis*—Erythromycin (3 µg/ml) + Ampicillin (100 µg/ml) + Carbenicillin (125 µg/ml). Bacterial CFUs were recorded and visualized using Microsoft Excel and R (R Core Team 2023) with RStudio (RStudio Team 2020). Graphical representation of CFUs was created using the *ggplot2* package (Wickham 2016).

### Biofilm biomass quantification

Overnight cultures of *P. koreensis* were diluted in 10% TSB to OD₆₀₀ of 0.004 (∼10^4^ CFU/ml), while *F. johnsoniae* and *B. cereus* were diluted to an OD₆₀₀ of 0.001 (∼10⁵ CFU/ml). Aliquots of the diluted cultures were transferred to 96-well plates (Sarstedt: 83.3924) and incubated at 18°C for 15 h. Overnight cultures of *P. aeruginosa* wild-type strains were diluted to a final OD₆₀₀ of 0.08 (∼10⁵ CFU/ml), and 5 µl of these cultures was added to each well. The plates were incubated for an additional 24 h at 18°C.

Biofilms were quantified by washing each well three times with 200 µl of 1 × PBS. To each well, 200 µl of 0.1% crystal violet (Sigma-Aldrich C0775) dissolved in 20% methanol:80% water was added, and biofilms were stained for 30 min. Wells were then washed once with 400 µl of 1 × PBS and twice with 200 µl.

The crystal violet stain was released by adding 200 µl of 33% acetic acid (Merck CAT#: 695 092). Absorbance was measured at 595 nm using a ThermoFisher Varioskan LUX microplate reader. Biofilm biomass was visualized using Microsoft Excel and R (R Core Team 2023) with RStudio (RStudio Team 2020), and graphical representation of the biofilm biomass were created using the *ggplot2* package (Wickham 2016).

## Results

### Invasion by *P. aeruginosa* reduces biofilm biomass

**Figure 1:**
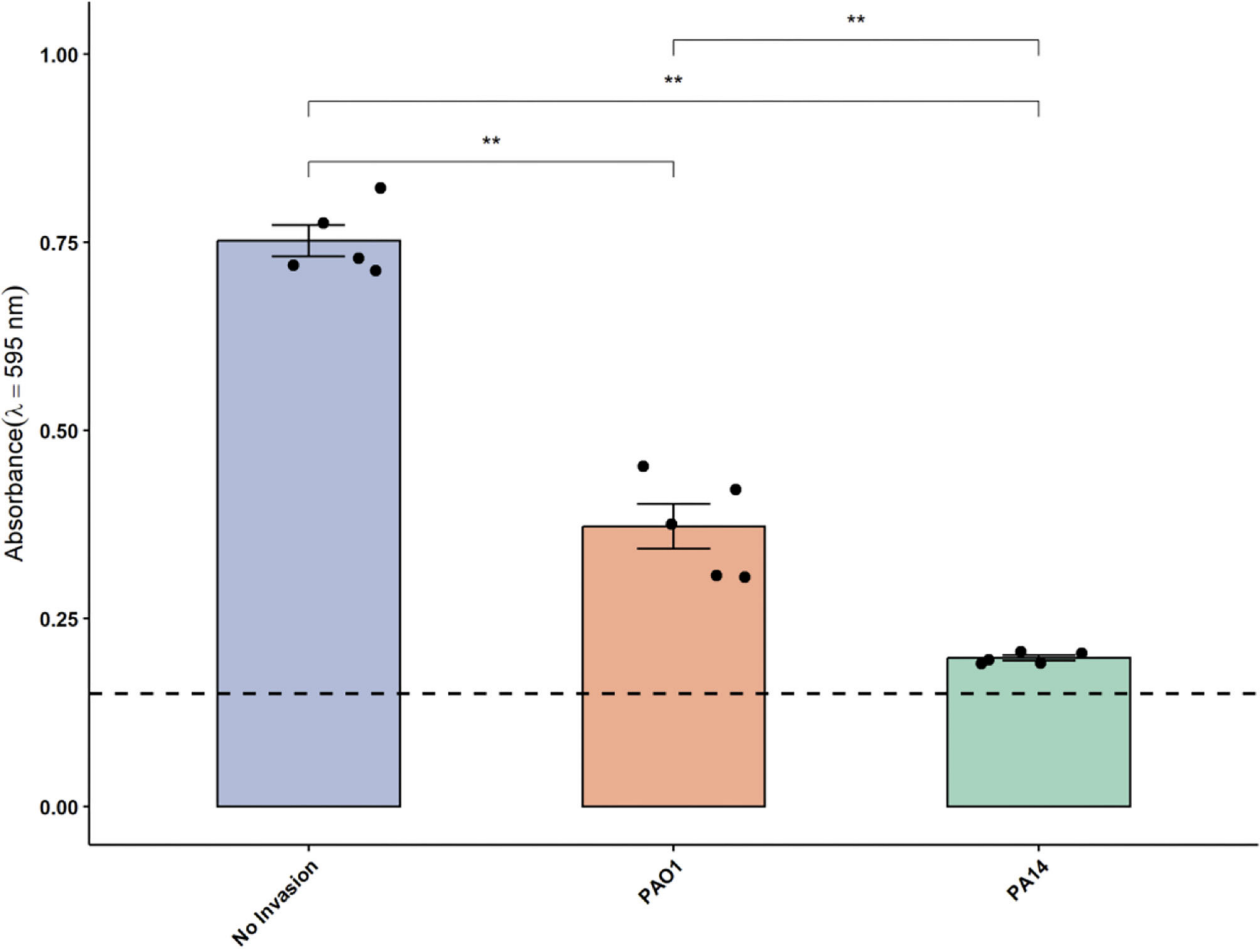
Effects of invasion by *P. aeruginosa* on the biofilm produced by THOR communities. Comparison between PAO1 strain and PA14 (n = 5, Student’s T-test *= P < 0.001*, * = *P* < 10^–6^). The dashed line represents the detection limit for biofilm biomass quantified by crystal violet staining. Error bars show the SEM, and each data point represents a biological replicate (n = 5), calculated as the average of its technical replicates (n = 8).

To investigate whether invasion with *Pseudomonas aeruginosa* influences the biofilm formation of established THOR communities, we compared the amount of biofilm produced by THOR when invaded by two *P. aeruginosa* strains, PAO1 and PA14, as well as with uninvaded THOR communities. A statistically significant decrease (Student’s t-test; *P* < 10^–7^) in biofilm biomass was observed in the THOR communities invaded by *P. aeruginosa* PAO1 (Fig. 1). Noticeably, we also found a significantly larger decrease when invaded by the virulent PA14 strain (Student’s t-test, *P* < 0.001; Fig. 1).

**Figure 2:**
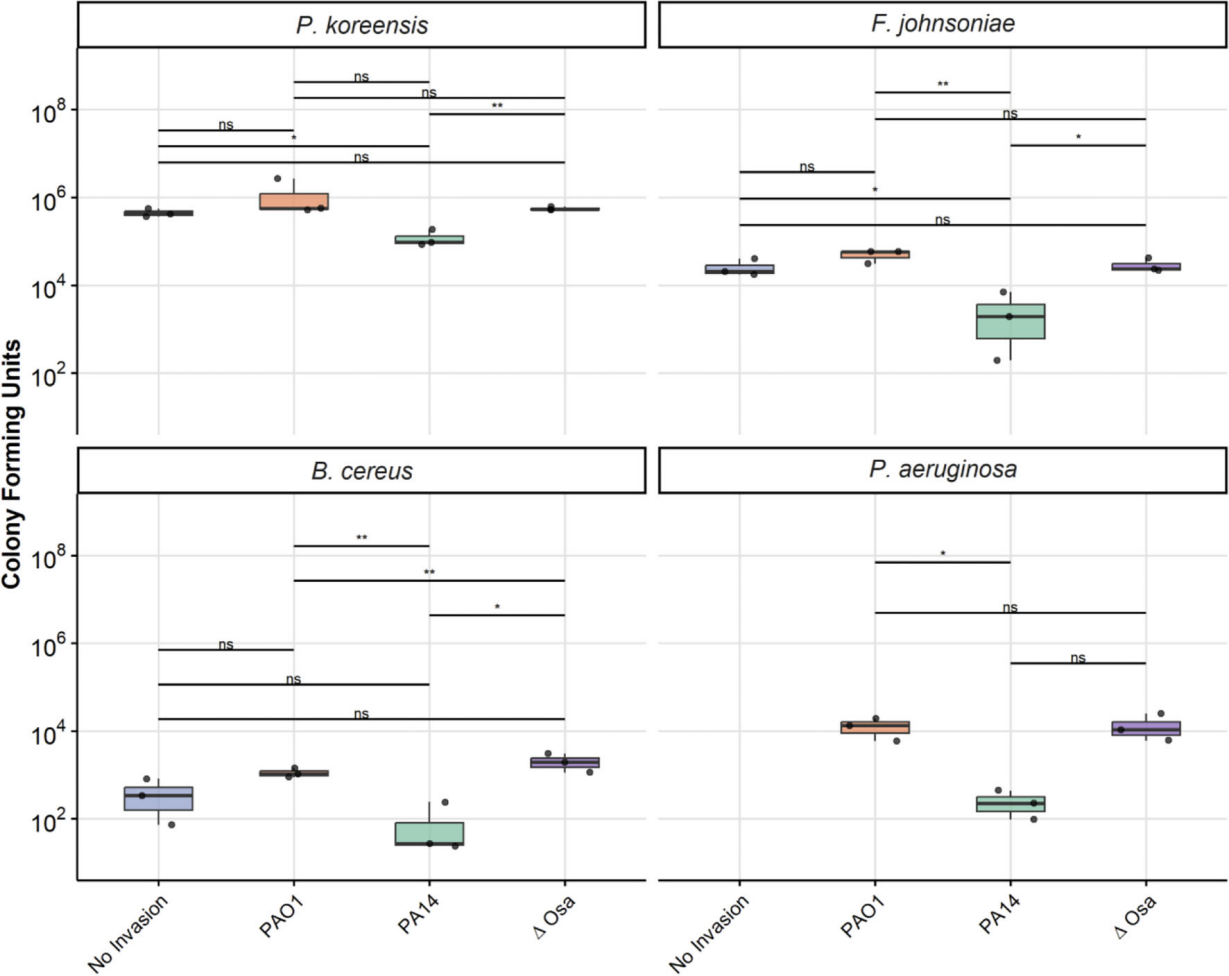
Impact of invaded THOR-biofilms on individual THOR community members. Impact of invaded THOR-biofilms on individual THOR community members. Data represent biological replicates (n = 3), with *P. aeruginosa* CFUs included where relevant. Both significant and non-significant statistical comparisons are shown, as they provide important context for interpreting the full pattern of invasion effects across conditions. Significance thresholds: *= P < 0.05*, * = *P* < 0.01.

This disruption led us to believe that there are competitive effects between both PAO1, PA14 and the THOR residents at a taxa level, so we moved forward by determining the abundance of individual strains from the invaded THOR biofilms. At taxa level, we discovered that invasion by PAO1 did not result in statistically significant changes of the abundances of community members within the biofilms, as measured as CFUs (Student’s t-test; *P* > 0.05; Fig. 2).

Noticeably, invasion by PA14 significantly decreased the CFU counts for *P. koreensis* and *F. johnsoniae. Pseudomonas koreensis* decreased from 5.6*10^6^/ml to 9.5*10^4^/ml, and *F. johnsoniae* from 7.8*10^4^/ml to 9.82*10^2^/ml. Furthermore, *B. cereus* decreased from 1.1*10^3^ to 8.2*10^2^; however, this was not a significant decrease (Fig. 2). Furthermore, an invasion with PAO1 wild type was perform, but no significant difference in colony counts compared to mPAO1 was observed (Fig S3.). This suggests that the disruption of THOR biofilm production may be dependent on the virulent background of the invading *P. aeruginosa* strain.

### Loss of O-antigen does not noticeably alter the competitive state of *P. aeruginosa*

During chronic *P. aeruginosa* pneumonia, there is selective pressure for the bacterium to shed one of its outermost antigens to evade the immune response (Demirdjian et al. 2017). These O-antigens play key roles in adhesion, environmental interaction, and, notably, virulence (Huszczynski et al. 2019). The loss of the O-antigen may provide a more accurate representation of *P. aeruginosa* in chronic pneumonia, suggesting that an absence of a functional O-antigen could be particularly relevant for examining competition between *P. aeruginosa* and microbial communities.The competitive loss could be due to the altered status of hydrophobicity of the lipopolysaccharide, reducing its adhesion onto the THOR biofilm (Murphy et al. 2014). Furthermore, when the O-antigen is lost there is a lost of integrity of the outer membrane is compromised, leading to a leaky membrane, which could further affect the attachment onto the biofilm (Huszczynski et al. 2019). To explore the impact of O-antigen loss on community invasion, we used a genetically engineered strain in which the chromosomal O-specific antigen (OSA) was deleted (Anbo et al. 2024).

We observed a statistically significant increase in *B. cereus* counts in THOR communities invaded by ΔOSA compared to PAO1 invaded communities (Fig. 2; Student’s t-test, *P* < 0.01). Furthermore, small increases were also detected in the populations of *F. johnsoniae* and *P. koreensis*. Notably, the abundance of *P. aeruginosa* was similar for ΔOSA strains and PAO1 while invading THOR. In contrast, mono-cultures of ΔOSA were significantly lower compared to wild-type (Fig. S2). These findings underscore the significant role of bacterial virulence factors in shaping the competitive dynamics between *P. aeruginosa* and the THOR microbial community, highlighting that virulence traits, typically associated with human hosts, also play ecological roles within microbial communities. In particular, the loss of O-antigen in *P. aeruginosa* notably impacted THOR. These results suggest that the absence of O-antigen may subtly influence competitive interactions within the microbial community, potentially altering *P. aeruginosa*’s invasive behavior and its competition for community resources.

To broaden our findings beyond engineered laboratory strains, we next investigated whether naturally occurring *P. aeruginosa* isolates differ in their interactions with the THOR community. We selected two clinical isolates representing distinct infection contexts: CCUG73475 from a patient with septic bacteremia and CCUG71613 from an intubated patient with chronic pneumonia. These isolates differ in their clinical histories and may vary in virulence traits, stress tolerance, and surface antigen expression. By comparing their invasion dynamics within THOR, we assessed whether these strain-specific characteristics translate into distinct ecological behaviors, offering insight into how clinical diversity shapes microbial competition.

**Figure 3:**
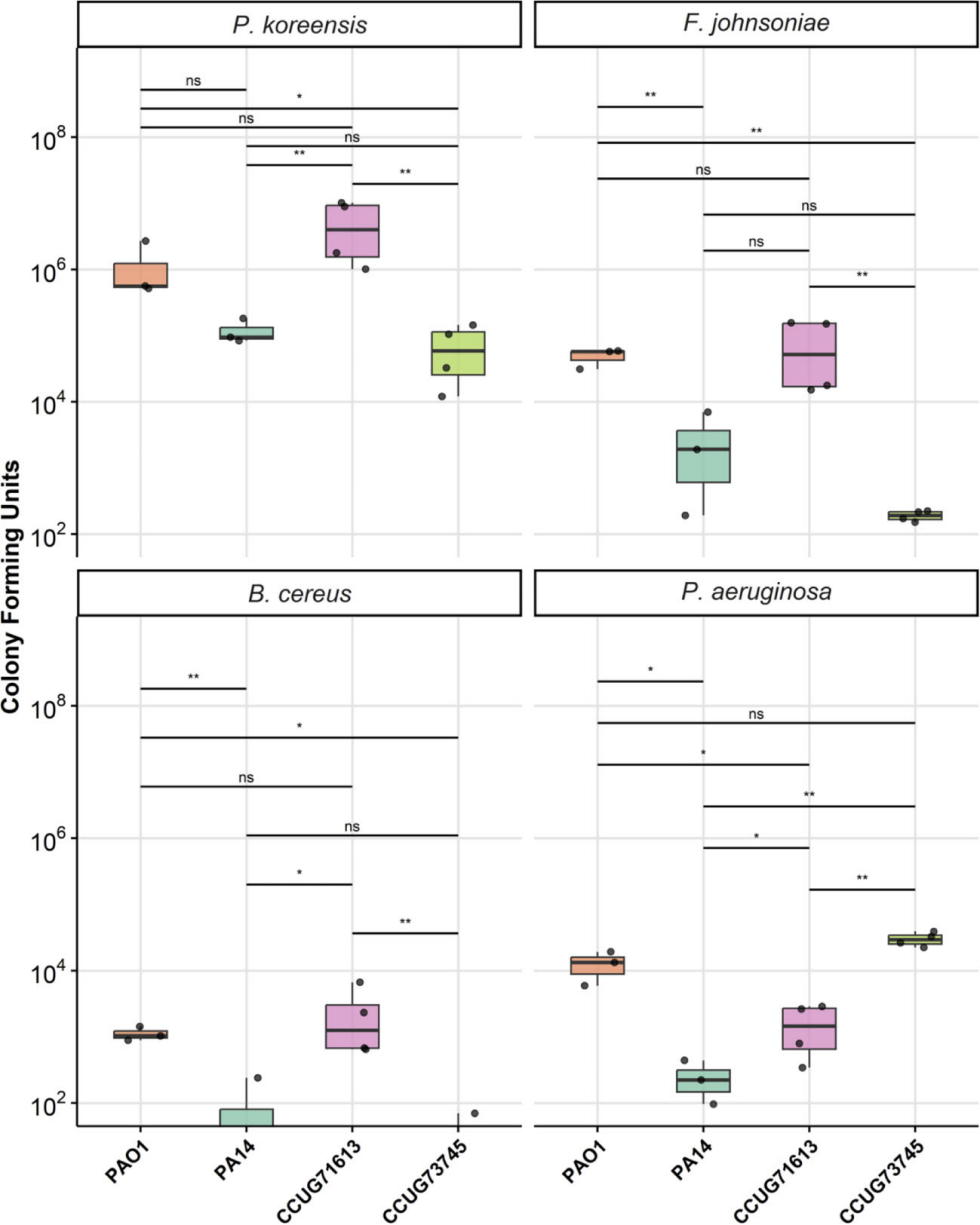
Impact of clinical *P. aeruginosa* isolates on individual THOR community members. THOR-biofilms were invaded with either the sepsis-derived isolate CCUG73475 or the pneumonia-derived isolate CCUG71613 and resulting changes in CFUs for each community member were quantified. Statistical significance was determined by Student’s T-test; * = *P* < 0.05, ** = *P* < 0.01.

Invasion with the pneumonia-derived isolate CCUG71613 resulted in minimal changes to the THOR community. CFU levels of *P. koreensis, F. johnsoniae*, and *B. cereus* remained comparable to those of invaded by PAO1, while a significant decrease was observed only in *P. aeruginosa* CFUs relative to PAO1-invaded communities. In contrast, invasion with the sepsis-derived isolate CCUG73475 led to a reduction in the CFU counts of all THOR community members, whereas *P. aeruginosa* CFUs for CCUG73475 remained at levels similar to those observed for PAO1(Fig. 3). These results demonstrate that the clinical isolates differ in their capacity to disrupt established THOR biofilms and in their resulting abundance within the invaded communities.

## Discussion

Our findings demonstrate that *P. aeruginosa* invasion of THOR biofilms significantly alters the biofilm production and microbial composition of the established communities, with those effects being dependent on the virulence profile of the invading strain. The more virulent PA14 strain caused a substantial reduction in biofilm biomass and significantly decreased the CFU counts of the resident community members, indicating strong competitive exclusion. In contrast, while PAO1 also disrupted biofilm formation, its impact on community structure was less pronounced. This suggests that the intensity of microbial competition is closely tied to the virulence potential of *P. aeruginosa*.

We observed a significant decrease in CFU counts for all isolated members of THOR biofilms, including *P. aeruginosa*, when invaded by the virulent strain PA14. This is congruent with the loss of total biofilm biomass during invasion, which we quantify using staining and measuring the absorbance resulting from the stained community biofilm of. When invaded with PA14, these absorbance values approach those of empty wells, suggesting substantial biofilm destruction. This implies that PA14 invasion reduces the available biofilm structure and the space that all community microbes can inhabit, which likely explains the lower colony counts observed for all members.

Multiple studies have shown that PAO1 and PA14 differ markedly in their virulence repertoires, with PA14 generally exhibiting a hyper-virulent phenotype compared to the moderately virulent PAO1 in diverse infection models (He et al. 2004, Lee et al. 2006, Mikkelsen et al. 2011, Grace et al. 2022). At the genomic level, PA14 carries several pathogenicity islands (PAPI-1, PAPI-2 and others) that are absent or only partially present in PAO1, and these encode additional virulence factors that contribute to its enhanced pathogenic potential (Lee et al. 2006, Harrison et al. 2010). A major functional difference lies in the Type III secretion system, where PAO1 expresses ExoS, ExoT and ExoY, whereas PA14 expresses the highly cytotoxic phospholipase ExoU instead of ExoS (Grace et al. 2022, Lee et al. 2023). ExoU-positive strains are associated with more severe host damage and rapid cell lysis, and may also influence interactions with neighbouring bacteria. Moreover, PAO1 and PA14 display distinct quorum-sensing regulation and biofilm strategies, including differences in exopolysaccharide usage, surfactant production and surface competition behaviour (Grace et al. 2022). Together, these strain-specific virulence traits provide a plausible mechanistic basis for the stronger disruption of THOR biofilms observed for PA14, for example through more aggressive contact-dependent antagonism and enhanced production of biofilm-destabilizing factors.

Virulence is a key determinant of bacterial competitive ability against hosts, as it influences both the aggressiveness of a bacterium and its ability to cause more severe damage, but may also increase competition between microbes (Kamiya et al. 2018). Highly virulent strains, like PA14, possess potent mechanisms—such as the secretion of toxins, enzymes, and antimicrobial compounds—that can inhibit or kill competing bacteria, colloquially referred to as bacterial warfare compounds (Granato et al. 2019, Booth et al. 2023). During community invasion, these strategies secure more resources and space for the invader, leading to significant reductions in the biomass and viability of resident microbial communities, as observed in our findings. Additionally, virulence factors can modulate the host immune response, creating an environment that favors the survival and proliferation of the virulent strain over less virulent competitors. This increasing competition between microbes may result in a selective pressure that would, in parallel, cross-select between virulence and inter-species competition.

In light of our data showing that *P. aeruginosa* strain PA14 invasion leads to marked declines in resident community biomass and diversity, it becomes clear that the very same virulence traits driving host damage are also at play in microbial competition. The secretion of exotoxins, degradative enzymes and secondary metabolites—traditionally studied for their roles in pathogenesis—appears to act also as an arsenal against neighboring bacteria, effectively reshaping community structure in favor of the invader. This duality helps explain why patients with greater microbiome disruption suffer worse clinical outcomes (Malhotra et al. 2019); interbacterial warfare compounds both undermine colonization resistance and exacerbate tissue injury. Moreover, by dampening or skewing host immune responses, virulence factors change the ecological balance, creating an environment where highly competitive—and highly virulent—strains prevail over less aggressive competitors. Together, these observations suggest a feedback loop in which selection for increased inter‐species competitiveness inherently selects for elevated virulence, and vice versa. Future work should therefore dissect the specific molecular effectors responsible for this cross‐selective pressure and explore whether targeting them can simultaneously mitigate pathology and preserve microbial community integrity.

Beyond virulence, structural adaptations, such as the loss of the O-antigen in *P. aeruginosa*, can also reshape competitive interactions within microbial communities. Although ΔOSA strains retain a similar overall competitive ability in monoculture, the absence of O-antigen alters their ecological performance within the THOR community, most notably by allowing *B. cereus* to flourish. This suggests that structural features modulate interactions such as resource competition and niche partitioning. Our data imply that O-antigen depletion favors *B. cereus* growth in the THOR community through at least two complementary mechanisms.

First, O-antigen is critical for surface adhesion and biofilm formation (Rocchetta et al. 1999, Murphy et al. 2014). Consistent with this, ΔOSA strains exhibited a ∼100-fold decrease in CFUs relative to wild-type when grown in monoculture biofilms (Fig. S1). Impaired adhesion limits biomass accumulation and prevents ΔOSA strains from reaching the cell densities required to activate density-dependent competitive and virulence pathways (Lee and Zhang 2015, Miranda et al. 2022).

Second, the structural properties of the O-antigen, particularly its length, modulate interactions with neighboring cells and influence the performance of secretion systems. Although O-antigen length has been linked to T3SS efficiency (Whitfield et al. 2020), the role of T3SS in direct microbial competition is considered largely indirect, and existing studies do not support a requirement for a fully functional T3SS in antagonism between human-associated microbes (Horna and Ruiz 2021). In contrast, the Type VI Secretion System (T6SS) is a well-established mediator of *P. aeruginosa* interbacterial competition, enabling contact-dependent delivery of antibacterial effectors (Basler et al. 2013, Sana et al. 2016, George et al. 2024). O-antigen loss may therefore alter competitive outcomes by increasing susceptibility to T6SS targeting or by impairing the ΔOSA strains’ ability to deploy its own T6SS machinery effectively, as O-antigen structure can influence cell–cell contact and effector delivery. These combined deficits would weaken *P. aeruginosa*’s competitive edge and allow *B. cereus* to proliferate within invaded biofilms.

A similar divergence in competitive behavior was observed when we examined the two clinical isolates. The pneumonia-derived strain CCUG71613 caused little disruption to the THOR community and even declined in abundance during invasion compared to invasion by PAO1, suggesting a competitive profile distinct from that of PAO1, PA14, or the sepsis isolate. Strains adapted to chronic airway infections frequently accumulate mutations that attenuate acute virulence functions while enhancing long-term persistence (Hogardt and Heesemann 2010), which may reduce their reliance on mechanisms causing antagonistic microbial interactions. In contrast, the sepsis-derived isolate CCUG73475 reduced the population sizes of all resident THOR members while maintaining its own CFU levels, reflecting a competitive strategy more similar to that of highly virulent or invasive *P. aeruginosa* lineages. These results indicate that the capacity for community disruption may ‘hitchhike’ on traits selected for during severe, invasive disease, where strong competitive and virulence mechanisms confer advantages both in the host and within microbial ecosystems. Together, the clinical isolates highlight how infection context and evolutionary history shape the competitive potential of *P. aeruginosa* beyond what can be inferred from laboratory reference strains alone.

Moreover, ΔOSA strains suffer a severe defect in propagating in the biofilms they create on their own. Despite this, it could be the case that they can exploit pre-existing THOR biofilms by hitchhiking on the attachment and extracellular polymeric substances produced by other community members. In this scenario, ΔOSA cells would colonize niches already created by the microbial community. Once embedded, even in low numbers, they might be able to piggyback on the structural scaffold, gaining access to nutrients and protection from shear forces without needing to build their own biofilm infrastructure. This hitchhiking mechanism would explain why ΔOSA strains show only a minor decrease in invasion into established THOR biofilms despite being profoundly impaired in de novo surface colonization.

Our model community, THOR, has inherent limitations. Notably, it cannot sustain continuous growth beyond 48 h due to nutrient depletion, restricting its ability to model long-term microbial dynamics. Continuous culture systems, such as those developed for studying cystic fibrosis infections (Foustoukos et al 2015, O’Brien and Welch 2019, Fan et al. 2019, Wang et al. 2022), provide a more robust platform for tracking microbial population shifts over extended periods. However, these models also lack key host-associated factors, such as adaptive immune responses and tissue-specific selective pressures, which shape microbial competition *in vivo*. The absence of these host factors limits the direct applicability of findings to infection contexts, where microbial interactions are further shaped by immune evasion and host-derived antimicrobial compounds.

Despite these constraints, our system effectively captures key aspects of *P. aeruginosa*’s competitive strategies, shedding light on the mechanisms driving its dominance in microbial communities. While THOR isolates are not derived from infected tissues (Boutin et al. 2017), the ability of *P. aeruginosa* to outcompete resident species and disrupt biofilm stability aligns with patterns observed in severe infections (Moradali et al. 2017, Frey et al. 2022, Quinn-Bohmann et al. 2024). Although the transferability of our results to host-associated infections remains uncertain, the microbial dynamics observed reinforce the broader role of *P. aeruginosa* as a dominant competitor across a multitude of microbiomes.

Taken together, our findings demonstrate that THOR is a valuable tool for studying pathogen-driven community disruption. By examining how *P. aeruginosa* with different virulence profiles influence microbial composition and antagonistic interactions, we highlight this system’s utility for dissecting invasion mechanisms. Understanding how both direct antagonistic mechanisms and broader ecological dynamics are connected to virulence traits is crucial for studying bacterial behavior and evolution, particularly in chronic infections where pathogens undergo adaptive changes to persist within microbial communities. Beyond this study, model communities like THOR can serve as useful tool sets for investigating broader microbial competition dynamics, offering insights into the ecological factors that drive pathogen success in diverse environments.

## Supplementary Material

fnag090_Supplemental_File
