# Supplementary material for: Virulent strains of Pseudomonas aeruginosa are more disruptive during invasion of a microbial model community
Source: FEMS Microbiol Lett. 2026 Jul 31;373:fnag090. doi: 10.1093/femsle/fnag090 (PMC13441163; doi:10.1093/femsle/fnag090)
Supplement: Supplementary file 1 [file fnag090_supplemental_file.docx]

**Figure S1. Colony forming units of isolates of *P. aeruginosa* biofilms which had been grown on empty surfaces.**

**Supplementary figure 2. Crystal violet formed by invadedTHOR communities by *P. aeruginosa* strains. (**=p<0.01, ****p<10^-6^ )**

**Supplementary figure 3. Isolates of THOR invaded by PAO1 and mPAO1 (n=3)**

**Table S1. Bacteria used in this work.**

| Bacterial strain: | Source | Publication |
| --- | --- | --- |
| PAO1 | Bacterial wounds infection | HOLLOWAY, B. W. (1955). Genetic Recombination in Pseudomonas aeruginosa. *Microbiology (Society for General Microbiology)*, *13*(3), 572–581. https://doi.org/10.1099/00221287-13-3-572 |
| mPAO1 | PAO1 clone | Jacobs, M. A., Alwood, A., Thaipisuttikul, I., Spencer, D., Haugen, E., Ernst, S., Will, O., Kaul, R., Raymond, C., Levy, R., Chun-Rong, L., Guenthner, D., Bovee, D., Olson, M. v, & Manoil, C. (2003). Comprehensive Transposon Mutant Library of Pseudomonas aeruginosa. *Proceedings of the National Academy of Sciences - PNAS*, *100*(24), 14339–14344. https://doi.org/10.1073/pnas.2036282100 |
| PA14 | Plant isolate | He, J., Baldini, R. L., Déziel, E., Saucier, M., Zhang, Q., Liberati, N. T., Lee, D., Urbach, J., Goodman, H. M., Rahme, L. G., & Nester, E. W. (2004). The Broad Host Range Pathogen Pseudomonas aeruginosa Strain PA14 Carries Two Pathogenicity Islands Harboring Plant and Animal Virulence Genes. *Proceedings of the National Academy of Sciences - PNAS*, *101*(8), 2530–2535. https://doi.org/10.1073/pnas.0304622101 |
| PAO1- Δosa (MA167) | Engineered PAO1 | Anbo M, Lubna MA, Moustafa DA, Paiva TO, Serioli L, Zor K, et al. (2024) Serotype switching in *Pseudomonas aeruginosa* ST111 enhances adhesion and virulence. PLoS Pathog 20(12): e1012221. https://doi.org/10.1371/journal.ppat.1012221 |
| *Pseudomonas koreensis* (CI12) | Co-isolate | Brook, P. S., K, D. A., K, K. A., & Jo, H. (2006). Peptidoglycan from Bacillus cereus Mediates Commensalism with Rhizosphere Bacteria from the Cytophaga-Flavobacterium Group. *Applied and Environmental Microbiology*, *72*(8), 5421–5427. https://doi.org/10.1128/AEM.02928-05 |
| *Flavobacterium johnsoniae* (UW101) | Co-isolate | J, M. M., & F, B. T. (2004). GldI Is a Lipoprotein That Is Required for Flavobacterium johnsoniae Gliding Motility and Chitin Utilization. *Journal of Bacteriology*, *186*(8), 2295–2302. https://doi.org/10.1128/jb.186.8.2295-2302.2004 |
| *Bacillus cereus* (UW85) | Soil-isolate | Handelsman J, Rafefel, S., Mester, E. H., Wunderlich, L., & Grau, C. R. (1990). Biological control of damping-off of alfalfa seedlings with Bacillus cereus UW85. *Applied and Environmental Microbiology*, *56*(3), 713–718. https://doi.org/10.1128/aem.56.3.713-718.1990 |
